# Combining professionalization and personalization in English long-term care: analyzing stakeholder views through a workforce lens

**DOI:** 10.3389/fsoc.2025.1719789

**Published:** 2026-01-13

**Authors:** Erika Kispeter, Shereen Hussein

**Affiliations:** ESRC Centre for Care, Department of Health Services Research and Policy, London School of Hygiene and Tropical Medicine, London, United Kingdom

**Keywords:** care quality, care workforce, healthcare quality, long-term care, personalization, person-centered care, policy, professionalization

## Abstract

**Introduction:**

Professionalizing the long-term care workforce, defined as improving the quality of care jobs, has been proposed as part of a solution to workforce challenges in long-term care. However, professionalization is argued to be in tension with personalization, a policy at the center of English long-term care. This article explores tensions and complementarities between the two policies through a workforce lens.

**Methods:**

We conducted qualitative group (*n* = 2) and one-to-one interviews (*n* = 7) with long-term care stakeholders (*n* = 25) representing a wide range of organizations in England. We have adopted the method of thematic analysis to explore stakeholders' views on the relationship between the professionalization of the hands-on care workforce and the personalization of care and support services.

**Results:**

We have identified three points of intersection between professionalization and personalization in stakeholders' narratives: care workers' autonomy, training and registration. Autonomy is defined here as care workers' discretion to make practical decisions in a care situation without the immediate approval of a manager or care professional. We have found that narratives reflected a complex relationship between the two policies. Stakeholders viewed care workers' autonomy and training as directly supporting the goals of personalization but they perceived personal assistants' formal training and registration as being in tension with personalization.

**Discussion:**

Care workers' practical autonomy emerged from our analysis of stakeholder narratives as a key aspect of improving care jobs (professionalization). This supports research findings that a higher degree of autonomy improves job satisfaction and it is a source of dignity in an undervalued occupation. Yet, autonomy is not explicitly included in definitions of professionalization in the context of English long-term care. This article contributes to the literature by conceptualizing care workers' autonomy as a dimension of professionalization, along with pay, terms and conditions of employment, training and registration. Secondly, the results contribute to the literature and to policy debates about the relationship between professionalization and personalization, two mechanisms of reforming long-term care systems globally. Our results demonstrate that there is a complex relationship between the two policy areas, characterized by synergies and tensions.

## 1 Introduction

Long-term care in England and globally is argued to be in crisis. The demand for care and support is growing, while the supply of formal services is severely impeded by chronic underfunding (Glasby et al., 2021). Nested in the broader crisis, there is a workforce crisis: difficulties in recruitment and especially in retention (Turnpenny and Hussein, 2020) have led to a severe shortage of care workers, a high turnover of staff and strong reliance on migrant workers (Hussein et al., 2024; Skills for Care, 2024a). Long-term care encompasses residential and home care services and community support for older people and working age people living with a physical or learning disability or mental health condition. Services include personal care (help with washing, dressing, eating and drinking) as well as support with accessing employment and community activities. The workforce includes registered professionals, such as social workers and nurses, but the majority are direct care workers (Skills for Care, 2024a), often referred to as “hands-on” or “front-line” staff. In this paper, the focus is on hands-on care workers, a group that includes personal assistants. Personal assistants may be self-employed but more commonly they are employed by the person they support, who uses their Personal Budget to pay for the services of the assistant. Personal assistants provide support with personal care but also with household tasks and attending appointments. They are more experienced and more satisfied with their jobs than the average care worker (Skills for Care, 2025).

Hands-on care work, like many jobs in the services economy, is considered to be “low-wage, low-trust, low-skill” (Milkman, 1998, p. 38). Indeed, care workers in England are low paid (Allen et al., 2025; Low Pay Commission, 2023), especially domiciliary care workers and personal assistants (Cominetti, 2023; Woolham et al., 2019). As for qualifications, more than half (56%) of front-line care staff in England do not have any form of long-term care qualification (Skills for Care, 2024b, Table 7.2) and there are currently no minimum requirements for formal qualifications in hands-on care jobs in England (Dodsworth and Oung, 2023).

As part of the solution to the workforce crisis, governments have taken action to “professionalize” the care workforce (see, for example, Curry et al., 2019; Kelly and Bourgeault, 2015; Scales, 2022). Similarly, in England several public bodies, such as the All Party Parliamentary Group on Adult Social Care (2019) and the Equality Human Rights Commission (2022), and care provider organizations (Care England, 2023; Homecare Association, 2022) have called for “professionalizing” the adult social care workforce, arguing that this would raise the status of long-term care work and thus contribute to easing the recruitment and retention challenges. As a concept applied to long-term care, professionalization is not clearly defined, however, the existing definitions include training, continuous professional development and compulsory registration of care workers as key dimensions (Hayes et al., 2019; Hemmings et al., 2022). In the devolved nations of the United Kingdom (Northern Ireland, Scotland and Wales) some elements of professionalization, such as compulsory minimum training, mandatory registration and pay rises have been introduced (Dodsworth and Oung, 2023; Hemmings et al., 2022), making England an outlier. Professionalization reforms have also been proposed in England: most recently the government's white paper, “People at the heart of care” (DHSC (Department of Health Social Care), 2021) outlined improved training and career pathways, however, the implementation of these policy proposals is delayed or stalled (Skills for Care, 2024c).

Professionalization is just one of a range of policies designed to reform long-term care in England. The relationship between different long-term care policies is increasingly the subject of scholarly research in the UK and internationally (Allen et al., 2023; Needham and Hall, 2023; Needham et al., 2023). Needham and Hall (2023) argue that professionalization and personalization are in tension conceptually, because they represent contrasting paradigms of long-term care: standardization and differentiation. Professionalization is argued to shift the long-term care system toward greater standardization through interventions such as the compulsory registration of care workers, while personalization (e.g., offering direct payments to people to purchase the care services they prefer) make the system more differentiated (Needham and Hall, 2023). Policy makers and practitioners pursue standardization and differentiation simultaneously and the unacknowledged policy tensions contribute to the lack of progress on long-term care reforms in England (Needham and Hall, 2023, p. 168).

To better understand the relationship between professionalization and personalization, we have analyzed long-term care stakeholders' narratives, exploring their views on the workforce implications of the two policy reforms. We make two contributions to the literature: we conceptualize care workers' autonomy as a dimension of professionalization, and we argue that despite representing contrasting paradigms of long-term care, professionalization and personalization can work together as policy goals. The rest of the introduction looks at the concepts of professionalization and personalization in long-term care and the literature on the how professionalization and personalization policies intersect in shaping the hands-on care workforce.

### 1.1 Conceptualizing the professionalization of the long-term care workforce

There are two main approaches to conceptualizing professionalization in the context of long-term care. The first approach (Hayes et al., 2019; Kremer, 2006) builds on the definition of a “professional”, a worker who has “individual decision-making responsibility”, who is “trusted to exercise personal judgement” and “supported with regular training to keep their skills and knowledge up-to-date” (Hayes et al., 2019, p. 1). Hayes et al. (2019) identify two key areas of professionalization in practice: mandatory registration and training, with the latter including induction. The authors emphasize that to achieve the aims of professionalization, care workers' pay and working conditions must also be improved at the same time (Hayes et al., 2019, p. 3).

Hayes et al. (2019) do not specify what level of qualifications would be sufficient for hands-on care workers, who are currently classified as performing “semi routine” work, to count as professionals. A professional is most commonly defined as belonging to a knowledge-based service occupation, having completed tertiary education and vocational training and accumulated experience (Evetts, 2006). There is a hierarchy of traditional or “real” professions, such as law and medicine and semi-professions, for example, nursing, teaching and social work that have their origins in vocational practice and are characterized by a less clearly defined body of knowledge, shorter training and less autonomy from supervision or societal control (Etzioni, 1969). It is argued that the gendering of occupations may explain why caring occupations, with majority female workforces are classified as semi-professions, in contrast to the traditionally male-dominated “real” professions (Dwyer, 2013; Evetts, 2003).

Rather than seeking to create classifications of “real” and “semi” professions, sociologists increasingly view differences between professions and other occupations as “differences of degree rather than kind” (Evetts, 2006, p. 134). Researchers have taken the same approach to studying the long-term care workforce: interpreting the “professional” character of different forms of care work to be on a continuum, with a formally qualified, employed and paid care worker at one end and an unqualified, unpaid and informal family carer at the other (Knijn and Verhagen, 2007; Kremer, 2006). Personal assistants are in an intermediary position: they are formally employed and paid, but their work is unregulated and they are not required to engage in formal training and professional development (Kremer, 2006).

Taking a different approach, Hemmings et al. (2022) conceptualize professionalization as a bundle of policies designed to improve the status of the long-term care workforce. Reviewing policies in the devolved nations of the UK and internationally, the authors identify the following dimensions of professionalization: registration and regulation; education, training and continuous professional development; pay and career progression; and terms and conditions of work (Hemmings et al., 2022, p. 9). This conceptualization encompasses more characteristics of care jobs and thus it is broader than that proposed by Hayes et al. (2019).

### 1.2 Conceptualizing the personalization of long-term care

Personalization is another hard-to-define concept, with a large body of literature discussing its different interpretations in policy discourse (Needham, 2011; Tarrant, 2020) and across different fields of health and care research, such as long-term care, dementia care and medicine (Wilberforce et al., 2017). Despite the lack of a precise definition, the policy goal to personalize long-term care services has become ubiquitous (Allen et al., 2023). Drawing on an extensive review of academic and policy literature, Ettelt et al. (2020) have identified two conceptualizations or two narratives of personalization: the first emphasizes people's ability to co-design their services and exercise choice and control over how services are provided and by whom, while the second, “personalized care”, emphasizes that personalization is an aspect of care and caring. It is important to note that while the two conceptualizations are analytically distinct, they are closely intertwined: both are relevant to all people drawing on care and support and all care workers.

The first narrative foregrounds the independence and autonomy of the person drawing on care. In England the choice and control narrative is closely linked to the disability movement's advocacy for breaking down the mass provision of care services and, at the same time, to the marketisation of adult social care, which constructs people drawing on care as “consumers” (Tarrant, 2020). “Choice and control” is closely linked to the mechanism of providing cash allocation to people drawing on publicly funded care, to the extent that access to personal budgets and direct payments is often used synonymously with the concept of personalization (Tarrant, 2020).

The second narrative of personalization, termed “personalized care”, is dominant in documents published by professional bodies such as the National Institute for Health and Care Excellence and the Care Quality Commission (Ettelt et al., 2020). It emphasizes the centrality of the care relationship to personalized care and care workers' ability to provide relationship-oriented care, and thus, “the role of the carer takes center stage, both individually through the act of providing care and as a member of a skilled workforce” (Ettelt et al., 2020, p. 52).

The two narratives foreground different aspects of personalization and they are argued to be in tension with one another (Ettelt et al., 2020; Wilberforce et al., 2017). This tension plays out in the design and delivery of care and support, for example, Ettelt et al. (2022) describe how care home managers tend to adopt one of these narratives when they talk about the services they provide. We expect that the stakeholders participating in our study will also use both narratives and this will influence how they view the relationship between personalization and professionalization.

### 1.3 The relationship between professionalization and personalization through a workforce lens

In this section we present evidence on how care workers and their practices create a link between professionalization and personalization. Policy documents make normative statements about personalization without specifying what personalized services should look like (Needham et al., 2016; Wilberforce et al., 2017) and without mentioning care workers (Lewis and West, 2014). However, the personalization agenda has created new expectations toward hands-on care workers—these can be summarized as “doing things with people rather than to them”. Hayes et al. (2019, pp. 10–11) emphasize the expectation that care workers should understand the principles of autonomy and self-determination and respect the autonomy of the people they support, for example, by co-creating solutions with people, rather than fixing problems for them. In terms of skills, it is argued that facilitating user-led support, the core idea of personalization, is a skill in itself that is closely linked to excellent communication and problem-solving skills (Hayes et al., 2019, pp. 13–14). Hayes and colleagues also emphasize that in the wake of personalization reforms hands-on care workers are now expected to have better decision making abilities and be more confident in “exercising personal judgement” (2019, p. 11).

Research with people who access care services has found that they want care workers, including personal assistants, to have the “right” personal qualities and values (Lewis and West, 2014; Walsh and Shutes, 2013) as well as relevant medical knowledge, for example, about different health conditions (Gridley et al., 2014; Moriarty et al., 2014). Perhaps unsurprisingly, people drawing on long-term care have different preferences for who provides care, with some choosing to employ friends and family as personal assistants while others prefer a more detached relationship with their care workers (Rodrigues, 2020).

Another body of research is focused on how care workers' behavior, knowledge, skills are relevant to personalization. Researchers have found that workers' emotional intelligence, values and adherence to ethical standards are of key importance to personalized care (Abrams et al., 2019; Schneider et al., 2019; Sutcliffe et al., 2021). Similarly, research has found that care workers' specialist knowledge, for example, about how dementias affect the behavior of the people they support can improve the quality of personalized care (Damant et al., 2023; Ettelt et al., 2020). Looking at training and education, there is evidence that interventions designed to develop care workers' emotional and interpersonal skills can improve the person-centeredness of care (Hayajneh and Shehadeh, 2014; Manthorpe et al., 2017) and more broadly, that training and experience can improve the quality of care (Allan and Vadean, 2021; Atkinson et al., 2018; Atkinson and Crozier, 2020; Crozier and Atkinson, 2024).

Research on care workers' skills, knowledge and training has also shown tensions with the goals of personalization. Hayes et al. (2019) note that the figure of a professional(ized), that is, trained, qualified and registered care worker suggests distance, rather than an equal relationship and power sharing that are the conditions of co-produced care and support. The authors aimed to alleviate this concern by arguing that “the professional care worker is not a ‘know-it-all' expert” but a worker who can “enable service-users in complex circumstances, with complex care or support needs” (2019, p. 6). However, there is evidence that some of those who employ personal assistants prefer workers without formal care qualifications or even work experience at a care provider company and instead they choose to train their assistants (Woolham et al., 2019). This does not mean that formal training is completely rejected, with Woolham et al. (2019) reporting that many personal assistants receive formal training after they have started work, with the consent and support of their employers. The strongest critique of professionalization from a personalization perspective relates to the idea of including personal assistants in the (proposed) mandatory register of care workers (Campbell, 2006; Gerlich and Farquharson, 2020). It is argued that registration would strengthen state control over the employment relationship between personal assistants and the people they assist (Graham et al., 2021), with critics emphasizing that this would contradict the ethos and goals of the independent living movement, a move away from paternalistic, state and expert-led services (Campbell, 2006).

The aim of this article is to explore how long-term care stakeholders in England make sense of the relationship between professionalization and personalization policies through a workforce lens, that is, exploring the implications of these policies for hands-on care workers. We analyze stakeholders' narratives along the research questions: What points of intersection between professionalization and personalization can be identified? Is professionalization seen to be supporting or hindering the personalization of care at these points of intersection? The rest of the article is structured as follows: in the next section we describe the research methods and in section three we present our analysis of stakeholders' narratives. In the final section we summarize our key findings, outline our arguments in full and discuss their relevance to the literature and long-term care policy.

## 2 Materials and methods

### 2.1 Design and data collection

This paper draws on data from a broader study entitled “Policy drivers of social care workforce change” that explored a range of policy reforms (including migration policies and the digitalization of social care) and their effects on the long-term care workforce in the UK's four nations (Hussein and Kispeter, 2025). Ethics approval was granted by the London School of Hygiene & Tropical Medicine's Observational/Interventions Research Ethics Committee (Reference 28339). Data collection took place between May 2023 and January 2024. Data generation methods for the broader study were as follows. We first conducted a scoping review of the literature on macro-level drivers of care workforce change. Then we conducted two online group interviews (with ten and nine participants respectively) and six one-to-one interviews, where participants commented on the findings of the literature review and discussed the workforce effects of policy change across the four nations of the UK. In the second phase of the study, we continued to work with the same participants and conducted a rapid prioritization exercise, two foresight (future scenario development) activities and a follow-up interview with one participant.

We adopted a purposive approach to selecting study participants: our aim was to include opinions from a wide variety of standpoints. The 25 participants represented a wide range of organizations with different and conflicting interests: long-term care regulatory bodies, associations of care provider organizations (employers), commissioners of services (local authority social services), care worker trade unions and organizations of older and disabled people and their informal carers who draw on social care. Other participants represented think tanks analyzing health and long-term care policy and advising policy makers. Information about stakeholder organizations is presented in Table 1.

**Table 1:** Study participants.

	**Organization**	**Country**	**Group/interview**
1	Think tank, health and long-term care policy	England	Group 1
2	Think tank, health and long-term care policy	England	Group 1
3	Think tank, social inequalities	England	Group 1
4	Care providers' association	England	Group 1
5	Think tank, long-term care policy	England	Group 1
6	Local authority social services	England	Group 1
7	Regulatory body, long-term care	Scotland	Group 1
8	Think tank, health and long-term care policy	England	Group 1
9	Regulatory body, long-term care	Scotland	Group 1
10	Charity supporting older people	England	Group 1
11	Regulatory body, long-term care	Scotland	Group 2
12	Think tank, workforce development in health and long-term care	England	Group 2
13	Trade union	England	Group 2
14	Professional organization, long-term care	England	Group 2
15	Regulatory body, long-term care	Wales	Group 2
16	Think tank, health and long-term care policy	England	Group 2 and interview
17	Care providers' association	England	Group 2
18	Organization supporting disabled people	England	Group 2
19	Academic researcher, long-term care	Northern Ireland	Group 2
20	Regulatory body, long-term care	Wales	Interview
21	Think tank, low pay and poverty	England	Interview
22	Trade union	England	Interview
23	Local authority social services	England	Interview
24	Think tank, workforce development in health and long-term care	England	Interview
25	Trade union	England	Interview

The analysis presented here is based on data from the two online group interviews and the seven one-to-one interviews. The group interviews were facilitated by both authors and lasted 90 min. The questions related to policy drivers of care workforce change; the implementation of policy reforms and their implications for the workforce; the relationship between different reform agendas and their effects on the care workforce. We shared the interview guides with the participants in advance to allow them time for preparation and set up an online whiteboard where participants could share ideas and reflections anonymously before and after the group interviews. We conducted one-to-one interviews with those stakeholders who were interested in participating but were unable to join the group discussions. The semi-structured interviews were focused on the same questions as the group discussions. They were conducted online by the first author and lasted 30–60 min. The follow-up interview, conducted by the first author in the second phase of the study was focused on professionalization policies.

In the group discussions, there was a lot of direct interaction between participants and they moved freely between the “live” discussion and the written chat. The chat function allowed participants to continue debates that have come to a close in the “live” discussion and to quickly respond to the ideas the speaker was expressing. The online whiteboard added an additional layer of data generation: as this was used by several participants before the group interviews, the comments written on the whiteboard were participants' initial ideas. The comments posted on the whiteboard were anonymous, unlike data generated in the interviews. Some of the whiteboard comments were long and detailed and included links to publications. The one-to-one interviews allowed stakeholders to give more detailed responses and the interviewer to ask follow-up and probing questions. In summary, the combination of one-to-one and group interviews and spoken and written (chat and the online whiteboards) formats generated rich data.

### 2.2 Analysis

The interviews were recorded with the informed consent of the participants. The recordings were transcribed verbatim and the text of the meeting chats and the online whiteboards was saved in separate documents and added to the dataset. We have analyzed three separate texts from each group discussion: the transcript of the audio-recording, the chat (written discussion) and the whiteboard. We have adopted thematic analysis (Braun and Clarke, 2006), a flexible but rigorous approach to analyzing the data. In the first wave of coding, conducted as part of the broader study, the first author analyzed the data deductively, applying codes based on the interview guides and the research questions of the broader study. When conducting the interviews and writing the research report (Hussein and Kispeter, 2025), it became clear that stakeholders held a variety of opinions on the relationship between professionalization and personalization and we decided to explore this issue.

The analysis for this article was focused only on the data (initial codes) that are related to both professionalization (the codes are: care workers' training, registration, pay, terms of employment, conditions of work and the status of care workers and the care sector) and personalization (person-centered care, tailored services, personal assistants, choice and control, direct payments and personal budgets). The first author continued the analysis, adopting a deductive approach based on the two research questions this paper aims to address. New codes were generated that reflected stakeholders' ideas about care workers' autonomy and the different aspects of training and skills development. The three points of intersection between professionalization and personalization, that is, the three key themes of the analysis relevant to both policy areas were agreed on.

The final stage of the analysis was focused on the relationship between the two policy areas. Taking an inductive approach, codes were generated that reflected the different interpretations of personalization, the interdependencies between different dimensions of professionalization and the complexities that relate to the employment of personal assistants. The two authors had regular discussions throughout the data generation and analysis, focusing on the emerging themes and the interpretation of the data. Addressing the first research question, we have identified three points of intersection between professionalization and personalization in stakeholder narratives: (1) care workers' autonomy at work, (2) training, and (3) registration. The next section is structured around these three themes.

## 3 Results

### 3.1 Care workers' autonomy at work

The first theme that emerged from the analysis as relevant to both professionalization and personalization was care workers' autonomy at work. Stakeholders used different terms to refer to the notion of autonomy, but they agreed that if care workers had (more) autonomy, they could better enact personalization. When talking about how personalization reforms were unfolding in England, Participant 12, representing a think tank that is focused on workforce development, noted that home care services could be more personalized if the managers of care provider organizations “listened more” to care workers and had more “confidence” in frontline staff's ability to personalize care (group 2). Another stakeholder responded to this idea and said that workers' lack of “agency” hindered the enactment of personalization:

Personalization is a lot about agency. To enable people to have that agency, we have to ensure that care workers have that agency themselves. … At times the jobs are framed as meaningless. ‘You are just a care worker, you have to call the office, you have to talk to the manager, the GP, the social worker.' They can't make many decisions and that makes it very difficult for personalization. It should be about the relationship between the person and the care worker. (P13, trade union, group 2)

This stakeholder called for care workers having more freedom to make decisions in a care situation without the supervision of a manager or a care professional. The quote depicts a domiciliary care worker who is with the person they support, trying to respond to an unanticipated situation or to meet the wishes of the person drawing on care. The stakeholder also emphasized that good care emerges from the care relationship and a care worker who cannot make decisions cannot co-produce care in a care situation.

Another participant, representing care provider organizations, agreed with the previous speaker that care workers needed more decision-making freedom and added that they were “the best people” to exercise personal judgement in a care situation, implying that care workers had a more in-depth knowledge of people they supported, rather than their managers. This participant used the term “autonomy” to express an idea similar to “agency”:

Care workers want and need some autonomy in their roles. They are the best people to judge, and they want that sense that they make a difference to people's lives. (P17, care providers' organization, group 2)

The three stakeholders quoted in this section did not use the word “professionalization”, however, it is clear that they called for better jobs, with more agency/autonomy and managers listening to staff. The second quote (Participant 13) explicitly contrasts the current low status of care workers and care jobs with a desired future higher status. In the third quote, Participant 17 suggests that having a degree of autonomy was also a source of job satisfaction that care workers wanted and needed.

In summary, stakeholders viewed autonomy as key to both higher status at work (professionalization) and more personalized care and support. It is important to note that autonomy, as conceptualized here is relevant only to workers who are employed by a care provider company and have a manager and it is not relevant to personal assistants, who are directly employed and managed by the person they support.

### 3.2 Training

Training and continuous professional development was the second point of intersection between the two policy agendas. Stakeholders viewed this dimension of professionalization as complementary with personalization, arguing that more highly trained care workers would be able to better enact professionalization. However, participants also acknowledged arguments (well known from public debates and research) that many people employing personal assistants view care workers' formal training to be in tension with personalization.

Several stakeholders told us that care workers increasingly used specialist clinical skills and knowledge as a growing number of people they supported had complex health as well as social care needs. Examples of tasks routinely performed in domiciliary settings included medication management, tube feeding and supporting people recovering from stroke. Staff received on-the-job training, the quality and depth of which varied across providers. A participant emphasized that care workers learned to perform clinical tasks and took on additional responsibilities without additional pay because they wanted to ensure that people received tailored, personalized care:

[Staff] will take on … clinical tasks … because it means that the person that they‘re supporting can stay at home and have this … procedure. … They can just live their normal life without having to go in the waiting room for an hour. (P24, arm's-length body, interview)

Some participants (for example, Participant 4, group 2) said that most employers trained their staff to do these tasks, but introducing formal qualifications would be useful. Others, however, argued that staff often lacked the necessary skills. In the following example, a stakeholder calls for (improved) training in end-of-life care:

We need to be training people to deliver the clinical care. … If we are honest about it, a lot of the care that's delivered in a care home is sort of palliative and end of life care and it's often delivered by people who don't have those particular skills. (P10, older people's charity, interview)

Stakeholders also talked about tensions between workforce training and personalization. A participant argued that people exercising choice and control over their support meant that care workers' so-called “soft skills” were increasingly emphasized in the care system and this de-emphasized the role of training: “There's a shift that takes away from the training, from medical skills toward soft skills, what the person wants” (Participant 6, local authority social services, group 1). She then added that “working age advocacy groups” were responsible for this shift:

There is tension, particularly from the working age adult advocacy groups, who say ‘I don't care about the qualifications, I want the soft skills. How the person [care worker] relates and treats me is what matters'. (P6, local authority social services, group 1)

Responding directly to these comments, another stakeholder noted that care workers' formal training encompassed the development of interpersonal skills as well:

This idea that there is a tension between the training element and personalization deserves more attention. Some people may see this [training] as a threat, it may reduce the pool of people, but there is evidence that training around person-centered care can be really beneficial. (P8, think tank, group 1)

The quote reveals that Participant 8 saw training as improving care workers' capacity to provide person-centered care, but she acknowledged that people drawing on care may oppose formal training. She added that the requirement of formal qualifications could also cause practical problems by reducing the supply of care workers in the labor market.

A third stakeholder, who represented an organization supporting disabled people, did not focus on tensions, rather, she talked approvingly about the efforts of some local authority social services departments to provide external support to personal assistants. She explained that these local authorities tried to “connect self-employed workers [personal assistants] … and implement quality assurance systems for them” (Participant 18, group 2). In other words, this stakeholder viewed training and workforce development, at least in a “light touch” form, as compatible with personalization.

Finally, many stakeholders talked about a different kind of tension when discussing training and workforce development: a tension between care workers' training and everyday practices of care. They argued that training was focused on person-centered care but in real life there were constraints that prevented workers from putting their skills and knowledge into practice. Participant 2 gave an example of a policy that limited care workers' opportunities to develop and utilize their skills and provide good person-centered skills:

In Scotland free personal care has created tension […] because it involves a more ‘one size fits all' approach. It likely has an impact on the skills required and the opportunities for developing these skills. (P2, think tank, group 1)

Participant 5 also pointed to the tension between training and everyday practices of care, referring to systemic constraints:

At the moment we have terms and conditions [of employment] set by commissioning processes, which can be very task-based … and then we have training that's care and outcome based. And then there may be an inspectorate, if you are looking at personalization, that's very risk averse. And it's not aligned. I think the care workforce feels pulled. (P5, think tank, group 1)

In other words, the participant suggested that currently workforce training was the most person-centered element of the care system, with the commissioning of services and inspections of care providers putting barriers to the enactment of personalization.

### 3.3 Registration

Care workers' occupational registration was the third point of intersection between professionalization and personalization that emerged from the data. Stakeholders discussed registration and its expected implications for the care workforce at length, but only a small part of the discussion was relevant to personalization. However, what they said was very important, with a stakeholder arguing that registration was in tension with personalization, specifically, that extending registration to personal assistants would limit people's options when recruiting their assistants:

There are clearly some areas of work where it's [registration] just not appropriate. The obvious one would be personal assistants. On what basis could you say to a working age adult with a disability ‘You can only employ someone from a register?' I mean, how could that ever make any sense? (P16, think tank, interview)

In the previous section we presented a similar quote, with Participant 6 highlighting that some people who drew on long-term care opposed formal training and qualifications for their support workers. When describing the policy tension, both stakeholders referred to “working age adults” the group of people whose interests would be negatively affected by professionalization.

Our analysis has revealed another way in which care workers' registration was seen relevant to personalization, albeit indirectly. Some stakeholders emphasized that registration would be an essential dimension of professionalization because it is intertwined with the training dimension, as the following quote demonstrates:

You actually professionalize the workforce by having some sort of system of registration, rather than some knowledge and skills framework, which is fine, but it's not really going to deliver anything concrete. You've got [to have] something really robust that you can sort of develop your training structures around. (P10, older people's charity, interview)

The “knowledge and skills framework” the participant criticized as not “robust” enough was proposed in the government's white paper entitled “People at the heart of care” [DHSC (Department of Health Social Care), 2021].

In summary, the key finding we reported in this subsection was a policy tension between personal assistants' registration and personalization. Stakeholders did not see registration as directly supporting the goals of personalization, however, an indirect relationship between registration and care workers' improved capacity to enact personalization also emerged from the analysis, with training seen as the mediator.

## 4 Discussion

This article aimed to explore the relationship between professionalization and personalization policies in English long-term care through a workforce lens, exploring the implications of these policies for hands-on care workers. We have analyzed stakeholder narratives along the research questions: What points of intersection between professionalization and personalization can be identified? Is professionalization seen to be supporting or hindering the personalization of care at these points of intersection?

Three points of intersection (themes) emerged from our analysis: care workers' autonomy, training, and registration. Autonomy is defined here as workers' ability to make decisions about care and support in a care situation without immediate permission or guidance from a care professional, for example, a social worker. By definition, this form of autonomy is not relevant to personal assistants, who are managed by the person they assist.

Addressing the second research question, we have found that stakeholder narratives reflected a complex relationship between the two policies. Autonomy was seen as supporting the goals of personalization: stakeholders argued that care workers with a higher degree of autonomy can better enact personalization. Training, both in clinical and interpersonal skills, was also seen as enabling care workers to better personalize care and support. However, stakeholders noted that some people employing personal assistants opposed the formal training of their assistants—stakeholders viewed this as a tension between the two policy areas. The mandatory registration of personal assistants was argued to be in tension with the goal of personalization, but some stakeholders viewed registration as a pre-requisite for improving training, which suggests that registration was seen as indirectly supporting the personalization of care. Lastly, we have found that when stakeholders focused on policy tensions, they adopted the “choice and control” narrative of personalization (Ettelt et al., 2020) that foregrounds people drawing on assistance and de-emphasizes care workers. When stakeholders focused on how autonomy and training support the aims of personalization, they adopted the “personalized care” narrative (Ettelt et al., 2020) that foregrounds the role of care workers in personalization.

Autonomy has emerged as a strong theme from our analysis. Stakeholders argued that care workers need a higher degree of autonomy than they currently have to utilize their person-centered skills in their everyday practice. It was also emphasized that a higher degree of autonomy would improve care workers' job satisfaction by making their jobs more meaningful. In other words, autonomy was seen as key both to making care and support more person-centered and to improving the paid work of care, that is the overarching aim of professionalization.

We first discuss autonomy in the context of professionalization. The views expressed by the stakeholders are in line with the results of empirical research on workers' autonomy as a characteristic of jobs. Researchers (Barken et al., 2018; Gleason et al., 2023) have found that autonomy is a key aspect of improving jobs: low-paid and unqualified care workers who have freedom to decide how to do their job experience higher levels of job satisfaction than those who do not have such autonomy. Stacey (2005) argues that autonomy is an important source of dignity in an occupation that is viewed as dirty and undervalued by the public.

The concept of workers' autonomy is well established in work and employment research (e.g., Lloyd and Payne, 2016), including research with long-term care workers (Hayes, 2017; Stacey, 2005). Autonomy at work is conceptualized as a dimension of job quality, where job quality is broadly defined as the characteristics of a job, such as pay and benefits, work organization, opportunities for training and skill development, participation and representation, and opportunities for career progression (Knox and Wright, 2022). Following Wardell (1992, quoted in Stacey, 2005), who distinguishes “practical” autonomy from “absolute” or “professional” autonomy, we emphasize that hands-on care workers' autonomy is practical autonomy. Such practical autonomy is essential in “high-touch” interactive service work where every encounter between the care worker and the “client” entails a high degree of unpredictability (Gatta et al., 2009).

Our first contribution is to the literature on professionalizing the long-term care workforce. Autonomy is not explicitly mentioned in current conceptualizations by Hayes et al. (2019) and Hemmings et al. (2022). Hayes et al. (2019, p. 1) refer to “decision-making responsibility/ability” as a characteristic of a professional worker and both sets of authors refer to the (desired) professional or “elevated” status of care workers and the workforce as a whole. We have adopted the term autonomy because it is well established in the literature on work and employment as a characteristic of all “good jobs” and not only of professions. We draw on Hemmings et al.'s (2022) conceptualization of professionalization as multi-dimensional and extend it by incorporating autonomy.

This article also contributes to the literature on the relationship between professionalization and personalization. The finding that workers' autonomy and skills development are seen as improving workers' capacity to enact personalization is in line with existing research on the quality of long-term care (Atkinson and Crozier, 2020; Burns et al., 2023) and specifically, the quality of person-centered care (Burns et al., 2016; Needham et al., 2016). Our result that stakeholders saw tensions between the two policies reiterates themes from long-standing debates about personal assistants' training and registration (Campbell, 2006;Davey, 2020; Gerlich and Farquharson, 2020).

The result that there is a complex relationship (both complementarities and tensions) between professionalization and personalization adds nuance to Needham and Hall's argument (2023) that the two policy areas are inherently in tension. What might explain this is, firstly, that we have taken a different approach to exploring the relationship between the policies: our analysis is focused on the hands-on care workforce and the everyday enactment of personalization while Needham and Hall (2023) contrast the vision of “good care” that is reflected in the policies. We have also disaggregated the dimensions of professionalization, while Needham and Hall (2023) discuss professionalizing the workforce as a one-dimensional concept.

Our results have relevance for long-term care policy and practice. We have found that care workers' autonomy is essential to both professionalization and personalization. However, the degree of autonomy that care staff can exercise in their day-to-day work cannot be increased in the same way as, for example, higher qualifications can be achieved by implementing a training program. Autonomy is created and recreated every day through the interactions between care workers, their supervisors, managers and care professionals. It is shaped by organizational human resource management, the broader workplace culture as well as the regulatory environment (Jacobsen et al., 2018; Crozier and Atkinson, 2024) and public discourses about long-term care. When care workers are first given a higher degree autonomy to make decisions with the people they care for, it is likely that they will need support, for example, peer support, as in the Buurtzorg model (Gray et al., 2015) of autonomous groups of workers making decisions as a team and supporting one another.

The stakeholders in our study emphasized that staff who have a degree of practical autonomy at work are more satisfied with their job and more motivated to continue to work in long-term care. However, granting care workers a higher degree of autonomy should not be used as a compensation for low pay, insecure employment and the lack of employee voice. Workers' self-exploitation is already a serious problem in the long-term care sector, with care workers doing unpaid work in their own time to provide personalized care (see, for example, Allard and Whitfield, 2024; Burns et al., 2016; Leverton et al., 2022), which contributes to their low levels of well-being (Hussein, 2018).

Oue discussion about autonomy raises questions about risk, responsibility and accountability. Hands-on care workers are already expected to balance the requirement to support people in making decisions for themselves and to help people stay safe (Carr, 2011; Hayes et al., 2019) but, as stakeholders argued, the care system is “risk averse”. For care workers to be able work autonomously with the people they support, it would be essential to clarify their roles, responsibilities and the boundaries around accountability. As (Glasby 2011, p. 11) argues, care workers will need support with positive risk taking “without fearing that we will come down on them like a ton of bricks if things go wrong”.

Echoing the participants of our study, we emphasize that interventions to professionalize the workforce do not necessarily translate into positive change for the workers or for the people they support, rather, the outcomes depend on the broader institutional context. Focusing on the outcomes for workers, researchers evaluating earlier professionalization efforts in England (Atkinson and Lucas, 2013; Gospel and Lewis, 2011) and the current wave of professionalization in Wales (Crozier and Atkinson, 2024) found that these policy interventions were focused too narrowly on training and lacked a “comprehensive” or “whole system” approach. Such an approach would include jobs with higher pay and routes to more senior or more specialist roles (career paths)—these are argued to be necessary for the benefits of skills development to be put into practice (Morrow et al., 2024). Looking at care quality, stakeholders in our study emphasized that high quality training in interpersonal skills in itself does not lead to more person-centered care and support, rather, workers need more autonomy and more time with the people they support to utilize their improved skills and co-produce care and support. In other words, the “time and task” model of “delivering” care should shift toward a more outcome-based model (Zimpel-Leal, 2021) and all elements of the system need to be “aligned”, for example, not only training but also care inspections should be focused on outcomes.

### 4.1 Limitations and areas for future research

There are four limitations to the study which require consideration. Firstly, in this article we have looked at only those dimensions of professionalization that are directly relevant to the goals of personalization and did not explore pay and terms of employment, conveying a narrow and somewhat utilitarian idea of professionalization. Improving the status of the long-term care workforce is an important aim in its own right, regardless of its impact on personalization. Secondly, though we present the views of stakeholders from a wide range of social care organizations, the stakeholders volunteered to participate in the study and their views do not represent all opinions. Closely related to this is the third limitation: the voice of care workers and people drawing on long-term care was represented in this study via trade unionists and advocacy organizations. Lastly, this article has focused exclusively on England and it has not explored stakeholders' views on the intersection of the two policy reforms in the other home nations of the United Kingdom where professionalization policies are being implemented and research about the intended and unintended consequences is emerging. This suggests future research focusing on the longer-term workforce implications of professionalization and personalization in these national contexts.

### 4.2 Conclusion

Policy arguments in favor of professionalization continue to surface in response to the long-standing workforce crisis and care workers' low pay and poor conditions of employment. For example, the Labor Government was elected in Summer 2024 on a manifesto promise to introduce a National Care Service in England that would improve and standardize training for care workers and introduce a system of career progression (Cooper and Harrop, 2023). However, personalization, a foundational policy of the long-term care system in England is widely perceived to be in tension with professionalization. This presents challenges to policy makers and those involved in putting policies into practice about how to combine the goals of personalization and professionalization.

This article has adopted a workforce lens to analyzing the views of long-term care stakeholders on the relationship between professionalization and personalization. Care workers' autonomy emerged as being key to both professionalization and personalization. We have found that the two policy agendas are not always in tension: workers' autonomy and training were understood as having the potential to improve the capacity of the care workforce to enact personalization. Based on these findings and drawing on the work and employment studies literature, we conceptualize care workers' autonomy as a separate dimension of professionalization.

The article contributes to research on professionalization and on interdependences and tensions between long-term care policies. Our findings contain learning for policy makers, commissioners and employers, whether they manage the staff of a care agency or employ a personal assistant. The findings suggest that there may be ways to combine the two policy agendas that benefit care workers and the people they support. Discussions about professionalization and personalization in long-term care would benefit from disaggregating the different dimensions of professionalization and different stakeholders should be more explicit about the competing narratives of personalization. This requires sensitivity to the values, interests and rights of both the people drawing on formal care services and those working in long-term care.

## Data Availability

The datasets presented in this article are not readily available because they are part of an ongoing study, and will be uploaded to a data archive at a later date. Requests to access the datasets should be directed to Erika Kispeter, erika.kispeter@lshtm.ac.uk.

## References

[B1] Abrams R. Vandrevala T. Samsi K. Manthorpe J. (2019). The need for flexibility when negotiating professional boundaries in the context of home care, dementia and end of life. Ageing Soc. 39, 1976–1995. doi: 10.1017/S0144686X18000375

[B2] All Party Parliamentary Group on Adult Social Care (2019). Elevation, Registration & Standardisation: The Professionalisation of Social Care Workers. London: APPG on Social Care. Available online at: https://creamhealthcare.co.uk/resources/news/the-professionalisation-of-social-care-workers/123/ (Accessed September 1, 2024).

[B3] Allan S. Vadean F. (2021). The Association between Staff Retention and English Care Home Quality. J. Aging Soc. Policy. 33, 708–724. doi: 10.1080/08959420.2020.1851349 33470916

[B4] Allard C. Whitfield G. J. (2024). Guilt, care, and the ideal worker: Comparing guilt among working carers and care workers. Gend. Work Organ. 31, 666–682. doi: 10.1111/gwao.12956

[B5] Allen K. Burn E. Hall K. Mangan C. Needham C. (2023). “They made an excellent start... but after a while, it started to die out”, tensions in combining personalisation and integration in English adult social care. Soc. Policy Soc. 22, 172–186. doi: 10.1017/S1474746422000392

[B6] Allen L. Vriend M. Naz A. Finch D. Alderwick H. (2025). Poverty, Pay and the Case for Change in Social Care. London: The Health Foundation. Available online at: https://www.health.org.uk/sites/default/files/upload/publications/2025/Poverty%20pay%20and%20the%20case%20for%20change%20in%20social%20care_FINAL-July.pdf(Accessed November 1, 2024).

[B7] Atkinson C. Crozier S. (2020). Fragmented time and domiciliary care quality. Employee Relat. 42, 35–51. doi: 10.1108/ER-05-2018-0142

[B8] Atkinson C. Crozier S. Lucas R. (2018). Workforce policy and care quality in English long-term elder care. Public Perform. Manag. Rev. 41, 859–884. doi: 10.1080/15309576.2018.1473784

[B9] Atkinson C. Lucas R. (2013). Worker responses to HR practice in adult social care in England. Hum. Res Mgmt J. 23, 296–312. doi: 10.1111/j.1748-8583.2012.00203.x

[B10] Barken R. Denton M. Sayin F. K. Brookman C. Davies S. Zeytinoglu I. U. (2018). The influence of autonomy on personal support workers' job satisfaction, capacity to care, and intention to stay. Home Health Care Serv. Q. 37, 294–312. doi: 10.1080/01621424.2018.1493014 30321126

[B11] Braun V. Clarke V. (2006). Using thematic analysis in psychology. Qual. Res. Psychol. 3, 77–101. doi: 10.1191/1478088706qp063oa

[B12] Burns D. Hamblin K. Fisher D. U. Goodlad C. (2023). Is it time for job quality? Conceptualising temporal arrangements in new models of homecare. Sociol. Health Illn. 45, 1541–1559. doi: 10.1111/1467-9566.13650 37191249

[B13] Burns D. J. Hyde P. J. Killett A. M. (2016). How financial cutbacks affect the quality of jobs and care for the elderly. Ind. Labor Relat. Rev. 69, 991–1016. doi: 10.1177/0019793916640491

[B14] Campbell J. (2006). Opinion: Registration Should Be a Matter of Choice, Community Care. Available online at: https://www.communitycare.co.uk/2006/11/02/opinion-registration-should-be-a-matter-of-choice/ (Accessed October 5, 2025).

[B15] Care England (2023). Care for Our Future: The Roadmap to a Sustainable Future for Adult Social Care. Available online at: https://www.careengland.org.uk/wp-content/uploads/2023/09/Care-for-Our-Future-Final.pdf (Accessed October 5, 2025).

[B16] Carr S. (2011). Enabling risk and ensuring safety: self-directed support and personal budgets. J. Adult Prot. 13, 122–136. doi: 10.1108/14668201111160723

[B17] Cominetti N. (2023). The Experience of Social Care Workers, and the Enforcement of Employment Rights in the Sector. Unbound and The Resolution Foundation. Available online at: https://www.resolutionfoundation.org/app/uploads/2023/01/Who-cares.pdf (Accessed April 20, 2023).

[B18] Cooper B. Harrop A. (2023). Support Guaranteed: The Roadmap to a National Care Service. London: Fabian Society. Available at: https://fabians.org.uk/wp-content/uploads/2023/06/Fabians-Support-Guaranteed-Report-WEB.pdf (Accessed October 5, 2025).

[B19] Crozier S. E. Atkinson C. (2024). ‘*You're only a care worker'*. Exploring the status of adult social care work through the intersection of HRM innovation and job quality. Int. J. Care Caring. 35, 1486–1511. doi: 10.1080/09585192.2023.2300033

[B20] Curry N. Schlepper L. Hemmings N. (2019). What Can England Learn from the Long-Term Care System in Germany? Nuffield Trust. Available online at: https://www.nuffieldtrust.org.uk/sites/default/files/2019-12/ltci-germany-br1924-6-web.pdf (Accessed October 5, 2025).

[B21] Damant J. Ettelt S. Perkins M. Lorraine W. Wittenberg R. Mays N. (2023). Facilitators of, and barriers to, personalisation in care homes in England: evidence from Care Quality Commission inspection reports. Int. J. Care Caring. 7, 91–113. doi: 10.1332/239788221X16426133095792

[B22] Davey V. (2020). Regulation of Care Workers - Should Personal Assistants be Included?. Social Care Institute for Excellence, 2020. Available online at: https://www.thinklocalactpersonal.org.uk/Blog/Regulation-of-care-workers-should-personal-assistants-be-included/ (Accessed October 5, 2025).

[B23] DHSC (Department of Health and Social Care) (2021). People at the Heart of Care: Adult Social Care Reform White Paper, CP 560, The Stationery Office. Available online at: https://www.gov.uk/government/publications/people-at-the-heart-of-care-adult-social-care-reform-white-paper (Accessed December 20, 2024).

[B24] Dodsworth E. Oung C. (2023). “What does the social care workforce look like across the four countries?,” in Adult Social Care in the Four Countries of the UK. Explainer series, Nuffield Trust. Available at: https://www.nuffieldtrust.org.uk/news-item/what-does-the-social-care-workforce-look-like-across-the-four-countries0 (Accessed December 20, 2024).

[B25] Dwyer R. E. (2013). The care economy? Gender, economic restructuring, and job polarization in the U.S. labor market. Am. Sociol. Rev. 78, 390–416. doi: 10.1177/0003122413487197

[B26] Equality and Human Rights Commission (2022). Experiences from Health and Social Care: The Treatment of Lower-Paid Ethnic Minority Workers. Available online at: https://www.equalityhumanrights.com/sites/default/files/2022/inquiry-experiences-and-treatment-of-lower-paid-ethnic-minority-workers-in-health-social-care-report.pdf (Accessed October 5, 2025).

[B27] Ettelt S. Damant J. Perkins M. Williams L. Wittenberg R. (2020). Personalisation in Care Homes for Older People. Policy Innovation and Evaluation Research Unit, London School of Hygiene and Tropical Medicine. Available online at: https://piru.ac.uk/assets/uploads/files/personalisation-in-care-homes-final-report.pdf (Accessed October 5, 2025).

[B28] Ettelt S. Williams L. Damant J. Perkins M. Wittenberg R. (2022). What kind of home is your care home? a typology of personalised care provided in residential and nursing homes. Ageing Soc. 42, 993–1013. doi: 10.1017/S0144686X20001142

[B29] Etzioni A. (1969). The Semi-Professionals and their Organization: Teachers, Nurses and Social Workers. New York: Free Press.

[B30] Evetts J. (2003). The construction of professionalism in new and existing occupational contexts: promoting and facilitating occupational change. Int. J. Sociol. Soc. Policy. 23, 22–35. doi: 10.1108/01443330310790499

[B31] Evetts J. (2006). Short note: the sociology of professional groups: new directions. Curr. Sociol. 54, 133–143. doi: 10.1177/0011392106057161

[B32] Gatta M. Boushey H. Appelbaum E. (2009). High-touch and here-to-stay: future skills demands in US low wage service occupations. Sociology. 43, 968–989. doi: 10.1177/0038038509340735

[B33] Gerlich K. Farquharson C. (2020, February 27). Experts' debate: will registering care workers reduce risk or restrict choice? The Guardian. Available online at: www.theguardian.com/society/2020/feb/27/experts-debate-registering-care-workers

[B34] Glasby J. (2011). Whose Risk is it Anyway? Risk and Regulation in an Era of Personalisation. York: Joseph Rowntree Foundation. Available online at: https://citizen-network.org/uploads/attachment/320/whose-risk-is-it-anyway.pdf (Accessed February 14, 2025).

[B35] Glasby J. Zhang Y. Bennett M. R. Hall P. (2021). A lost decade? a renewed case for adult social care reform in England. J. Soc. Pol. 50, 406–437. doi: 10.1017/S0047279420000288

[B36] Gleason H. P. Miller E. A. Boerner K. (2023). Focusing on the positive: Home health aides' desire for autonomy and control. J. Appl. Gerontol. 42, 728–736. doi: 10.1177/07334648221145177 36523133

[B37] Gospel H. Lewis P. A. (2011). Who cares about skills? the impact and limits of statutory regulation on qualifications and skills in social care. Br. J. Ind. Relat. 49, 601–622. doi: 10.1111/j.1467-8543.2010.00828.x

[B38] Graham K. Brooks J. Maddison J. Birks Y. (2021). Two jobs in one day: exploring the dynamics of personal assistance relationships in the workplace. Scand. J. Disabil. Res 23, 147–157. doi: 10.16993/sjdr.761

[B39] Gray B. H. Sarnak D. O. Burgers J. S. (2015). Home Care by Self-Governing Nursing Teams: The Netherlands' Buurtzorg Model. New York, NY United States: Commonwealth Fund. doi: 10.15868/socialsector.25117

[B40] Gridley K. Brooks J. Glendinning C. (2014). Good practice in social care: the views of people with severe and complex needs and those who support them. Health Soc. Care Community. 22, 588–597. doi: 10.1111/hsc.12105 24697946

[B41] Hayajneh F. A. Shehadeh A. (2014). The impact of adopting person-centred care approach for people with Alzheimer's on professional caregivers' burden: an interventional study. Int. J. Nurs. Pract. 20, 438–445. doi: 10.1111/ijn.12251 25039325

[B42] Hayes L. (2017). Stories of Care: A Labour of Law, London, UK: Palgrave. doi: 10.1057/978-1-137-49260-9

[B43] Hayes L. Johnson E. Tarrant A. (2019). Professionalisation at Work in Adult Social Care: Report to the All-Party Parliamentary Group on Adult Social Care. Unison. Available online at: https://kar.kent.ac.uk/77269/1/Professionalisation_at_Work_0309.pdf (Accessed June 1, 2024).

[B44] Hemmings N. Oung C. Schlepper L. (2022). New Horizons: What can England Learn from the Professionalisation of Care Workers in Other Countries? Available online at: https://www.nuffieldtrust.org.uk/research/new-horizons-what-can-england-learn-from-the-professionalisation-of-care-workers-in-other-countries (Accessed June 1, 2023).

[B45] Homecare Association (2022). Homecare Association Response to Regulation of Professionals Consultation. Consultation submission, 31st March 2022. Available online at: https://www.homecareassociation.org.uk/resource/regulation-of-professionals-pdf.html (Accessed October 5, 2025).

[B46] Hussein S. (2018). Job demand, control and unresolved stress within the emotional work of long-term care in England. Int. J. Care Car. 2, 89–107. doi: 10.1332/239788218X15187915863909

[B47] Hussein S. Kilkey M. Tawodzera O. (2024). The vulnerability of Central & Eastern European and Zimbabwean migrant home care workers' wellbeing in the UK: the intersectional effects of migration and social care systems. J. Ethn Migr. Stud. 50, 1118–1137. doi: 10.1080/1369183X.2023.2279716

[B48] Hussein S. Kispeter E. (2025). Policy Drivers of Social Care Workforce Change: United Kingdom Insights, Impacts, and Future Directions. Sheffield: CIRCLE, University of Sheffield. Available online at: https://centreforcare.ac.uk/wp-content/uploads/2025/09/SH-EK-Research-Report-May-2025-FINAL.pdf (Accessed October 5, 2025).

[B49] Jacobsen F. F. Day S. Laxer K. Lloyd L. Goldmann M. Szebehely M. . (2018). Job autonomy of long-term residential care assistive personnel: a six country comparison. Ageing Int. 43, 4–19. doi: 10.1007/s12126-017-9291-9

[B50] Kelly C. Bourgeault I. (2015). The personal support worker program standard in Ontario: an alternative to self-regulation? Hcpol. 11, 20–26. doi: 10.12927/hcpol.2016.24450 26742113 PMC4729280

[B51] Knijn T. Verhagen S. (2007). Contested professionalism: payments for care and the quality of home care. Adm. Soc. 39, 451–475. doi: 10.1177/0095399707300520

[B52] Knox A. Wright S. (2022). “Understanding job quality using qualitative research,” in The Oxford Handbook of Job Quality, eds. Warhurst C. Mathieu C. Dwyer R. E. (Oxford: Oxford University Press), 107–125. doi: 10.1093/oxfordhb/9780198749790.013.6

[B53] Kremer M. (2006). Consumers in charge of care: the Dutch personal budget and its impact on the market, professionals and the family. Eur. Soc. 8, 385–401. doi: 10.1080/14616690600822006

[B54] Leverton M. Samsi K. Woolham J. Manthorpe J. (2022). Lessons learned from the impact of Covid-19 on the work of disability support organisations that support employers of social care personal assistants in England. Health Soc. Care Community 30:e6708–e6718. doi: 10.1111/hsc.14098 36345869 PMC9877777

[B55] Lewis J. West A. (2014). Re-shaping social care services for older people in England: Policy development and the problem of achieving “good care.” J. Soc. Policy. 43, 1–18. doi: 10.1017/S0047279413000561

[B56] Lloyd C. Payne J. (2016). Skills in the Age of Over-Qualification: Comparing Service Sector Work in Europe, 1st ed. Oxford: Oxford University press. doi: 10.1093/acprof:oso/9780199672356.001.0001

[B57] Low Pay Commission (2023). Low Pay Commission Report 2022. Available online at: https://www.gov.uk/government/publications/low-pay-commission-report-2022 (Accessed December 20, 2024).

[B58] Manthorpe J. Harris J. Samsi K. Moriarty J. (2017). Doing, being and becoming a valued care worker: user and family carer views. Ethics Soc. Wel. 11, 79–91. doi: 10.1080/17496535.2016.1247904

[B59] Milkman R. (1998). “The new American workplace: high road or low road?,” in Workplaces of the Future, eds. Thompson P. Warhurst C. (Basingstoke: Macmillan), 25–39. doi: 10.1007/978-1-349-26346-2_2

[B60] Moriarty J. Manthorpe J. Cornes M. (2014). Skills social care workers need to support personalisation. Soc. Care and Neurodisabil. 5, 83–90. doi: 10.1108/SCN-12-2013-0042

[B61] Morrow E. Kelly C. Killeen C. Naessens E. Lynch M. (2024). Exploring a career pathway for home support workers in Ireland: a systematic scoping review of the international evidence. Front. Health Serv. 4:1360920. doi: 10.3389/frhs.2024.1360920 38545381 PMC10967662

[B62] Needham C. (2011). Personalization: From story-line to practice. Soc. Policy Adm. 45, 54–68. doi: 10.1111/j.1467-9515.2010.00753.x

[B63] Needham C. Allen K. Hall K. (2016). “Enacting personalization on a micro scale,” in Micro-Enterprise and Personalization: What Size is Good Care?, eds. Needham C. Allen K. Hall K. (Bristol: Bristol University Press), 111–128. doi: 10.51952/9781447319245.ch007

[B64] Needham C. Foster M. Fisher K. R. Hummell E. (2023). Tailored and seamless: Individualised budgets and the dual forces of personalisation and Collaboration. Soc. Policy Soc. 22, 127–138. doi: 10.1017/S1474746422000434

[B65] Needham C. Hall P. (2023). Social care in the UK's Four Nations: Between Two Paradigms. Bristol: Policy Press. ISBN: 978-1447364641. doi: 10.1332/policypress/9781447364641.001.0001

[B66] Rodrigues R. (2020). Caring relationships and their role in users' choices: a study of users of direct payments in England. Ageing Soc. 40, 1469–1489. doi: 10.1017/S0144686X19000035

[B67] Scales K. (2022). Transforming direct care jobs, reimagining long-term services and supports. J. Am. Med. Dir. Assoc. 2, 207–13. doi: 10.1016/j.jamda.2021.12.005 34973168

[B68] Schneider J. Pollock K. Wilkinson S. Perry-Young L. Travers C. Turner N. (2019). The subjective world of home care workers in dementia: an “order of worth” analysis. Home Health Care Serv. Q. 38, 96–109. doi: 10.1080/01621424.2019.1578715 30794075

[B69] Skills for Care (2024a). The State of the Adult Social Care Sector and Workforce in England 2024. Available online at: https://www.skillsforcare.org.uk/Adult-Social-Care-Workforce-Data/Workforce-intelligence/publications/national-information/The-state-of-the-adult-social-care-sector-and-workforce-in-England.aspx (Accessed October 5, 2025).

[B70] Skills for Care (2024b). Workforce Estimates – Table 7.2. Available online at: https://www.skillsforcare.org.uk/Adult-Social-Care-Workforce-Data/Workforce-intelligence/publications/Workforce-estimates.aspx (Accessed December 20, 2024)

[B71] Skills for Care (2024c). A Workforce Strategy for Adult Social Care in England. Available online at: https://www.skillsforcare.org.uk/Workforce-Strategy/Home.aspx (Accessed October 5, 2025).

[B72] Skills for Care (2025). Individual Employers and the Personal Assistant Workforce. Skills for Care. Available online at: https://www.skillsforcare.org.uk/Adult-Social-Care-Workforce-Data/workforceintelligence/resources/Reports/Topics/IE-PA-report-2025.pdf (Accessed October 5, 2025).

[B73] Stacey C. L. (2005). Finding dignity in dirty work: the constraints and rewards of low-wage home care labour. Sociol. Health Illn. 27, 831–854. doi: 10.1111/j.1467-9566.2005.00476.x 16283901

[B74] Sutcliffe C. Davies K. Ahmed S. Hughes J. Challis D. (2021). Delivering personalized home care for people with dementia: an investigation of care providers' roles and responsibilities. J. Long Term Care. 58–69. doi: 10.31389/jltc.35

[B75] Tarrant A. (2020). Personal budgets in adult social care: The fact and the fiction of the Care Act 2014. J. Soc. Welfare Fam. Law. 42, 281–298. doi: 10.1080/09649069.2020.1796224

[B76] Turnpenny A. Hussein S. (2020). Recruitment and Retention of the Social Care Workforce: Longstanding and Emerging Challenges during the COVID-19 Pandemic A Research Brief . Available at: https://researchonline.lshtm.ac.uk/id/eprint/4662686/ (Accessed October 5, 2025).

[B77] Walsh K. Shutes I. (2013). Care relationships, quality of care and migrant workers caring for older people, Ageing Soc. 12: 2, 205–19.

[B78] Wilberforce M. Challis D. Davies L. Kelly M. P. Roberts C. Clarkson P. (2017). Person-centredness in the community care of older people: a literature-based concept synthesis, Int. J. Soc. Welfare. 26, 86–98. doi: 10.1111/ijsw.12221

[B79] Woolham J. Norrie C. Samsi K. Manthorpe J. (2019). Roles, Responsibilities, and Relationships: Hearing the Voices of Personal Assistants and Directly Employed Care Workers. London: King's College London.

[B80] Zimpel-Leal K. (2021). “Emergent homecare models are shaping care in England: an ethnographic study of four distinct homecare models,” in The Contributions of Health Care Management to Grand Health Care Challenges, eds. Hefner J. L. Nembhard I. M. (Leeds, UK: Emerald Publishing Limited), 3–27. doi: 10.1108/S1474-823120210000020001 34779190

